# Mitogenome Characteristics and Intracellular Gene Transfer Analysis of Four *Adansonia* Species

**DOI:** 10.3390/genes16070846

**Published:** 2025-07-21

**Authors:** Tingting Hu, Fengjuan Zhou, Lisha Wang, Xinwei Hu, Zhongxiang Li, Xinzeng Li, Daoyuan Zhou, Hui Wang

**Affiliations:** 1 Forestry College, Xinyang Agriculture and Forestry University, Xinyang 464000, China; 2 Biyang County Forestry Technology Work Station, Zhumadian 463700, China; 3 Xincai County Forestry Development Service Center, Zhumadian 463500, China; 4 Shangcai County Forestry Development Service Center, Zhumadian 463800, China; 5 Fuzhuang Township Government Law Enforcement Brigade, Zhumadian 463712, China

**Keywords:** *Adansonia*, mitogenome, intracellular gene transfer, phylogeny

## Abstract

*Adansonia* L. (1753) belongs to the family Malvaceae and is commonly known as the baobab tree. This species holds significant cultural and ecological value and is often referred to as the ‘tree of life.’ Although its nuclear genome has been reported, the mitogenome has not yet been studied. Mitogenome research is crucial for understanding the evolution of the entire genome. In this study, we assembled and analyzed the mitogenomes of four *Adansonia* species by integrating short-read and long-read data. The results showed that the mitogenomes of all four *Adansonia* species were resolved as single circular sequences. Their total genome lengths ranged from 507,138 to 607,344 bp and contained a large number of repetitive sequences. Despite extensive and complex rearrangements between the mitogenomes of *Adansonia* and other Malvaceae species, a phylogenetic tree constructed based on protein-coding genes clearly indicated that *Adansonia* is more closely related to the *Bombax*. Selection pressure analysis suggests that the *rps4* gene in *Adansonia* may have undergone positive selection compared to other Malvaceae species, indicating that this gene may play a significant role in the evolution of *Adansonia*. Additionally, by analyzing intracellular gene transfer between the chloroplast, mitochondria, and nuclear genomes, we found that genes from the chloroplast and mitochondria can successfully transfer to each chromosome of the nuclear genome, and the *psbJ* gene from the chloroplast remains intact in both the mitochondrial and nuclear genomes. This study enriches the genetic information of *Adansonia* and provides important evidence for evolutionary research in the family Malvaceae.

## 1. Introduction

*Adansonia* L. 1753, commonly known as baobabs, is an iconic genus of trees in the family Malvaceae, celebrated as the ‘trees of life’ for their massive trunks, remarkable longevity, and profound cultural and ecological significance [1,2]. The genus comprises eight species [3,4], six of which are endemic to Madagascar (e.g., *Adansonia grandidieri* and *Adansonia perrieri*). One species (*Adansonia digitata*) is widely distributed across Africa, while another (*Adansonia gregorii*) is exclusively found in north-west Australia. According to the IUCN Red List, *A. perrieri* in Madagascar is classified as Critically Endangered, while *A. grandidieri* and *Adansonia suarezensis* are listed as Endangered [5]. Although the remaining species are currently classified as non-threatened, their populations are declining, necessitating urgent conservation efforts. Baobabs are quintessential multi-purpose trees, with their fruit, leaves, bark, and other parts being used extensively by local communities. The fruit is rich in vitamin C, antioxidants, and minerals, and its pulp is often processed into beverages or dried foods, serving as a vital emergency resource during famines [6,7,8]. The leaves can be eaten as vegetables or used for medicinal purposes, while the bark fibers can be used for weaving and in construction [9]. Beyond their socio-economic value, baobabs play a pivotal role in ecosystems by providing critical habitat for wildlife, enhancing soil fertility, and contributing significantly to carbon sequestration [9]. In the context of an ageing society and the increasing prevalence of metabolic diseases such as diabetes, the potential health benefits of *A*. *digitata* fruit and its role in blood glucose control have garnered significant attention. Research indicates that its extracts may exert anti-diabetic effects by regulating insulin signaling pathways and the activity of glucose transporters [10]. Therefore, in-depth studies of *Adansonia*’s genomics, chemical composition, and physiological functions not only contribute to enriching its genetic information but also provide a scientific basis for its applications in food, pharmaceuticals, and ecological conservation.

Recent studies have used high-quality chromosome-level genome sequencing to reveal the genetic diversity and evolutionary history of baobabs [5]. Genome-wide analyses show that the chromosome base number (n = 44) is relatively conserved across all diploid species [11]. However, a chromosome fusion event has led to *A. perrieri* having a chromosome number of n = 42 [11]. The African tetraploid species, *A. digitata*, has been identified as an autopolyploid [12]. Its genome retains ancestral traits that might have promoted adaptation and expansion across the African continent via polyploidization [5]. Phylogenetic studies support Madagascar as the evolutionary center of extant baobab trees. Species divergence started about 20 million years ago, which is much later than the Gondwana continental split [5]. This suggests that transoceanic dispersal was crucial for the formation of species distribution patterns. Gene flow analyses have revealed ancient genetic exchanges between Malagasy species and *A. gregorii* in Australia, as well as *A. digitata* in Africa, indicating possible historical geographic overlap. Moreover, persistent interbreeding among intra-island species (e.g., *A. za* and *A. perrieri*) has further increased genetic complexity [5]. Plant mitogenomes are important genetic material that is responsible for energy metabolism in plant cells [13]. They have unique structural features and significant research value. Compared with animal mitogenomes, plant mitogenomes exhibit significant differences in structure, function, and evolution [14,15]. Their genome sizes are usually much larger than those of animals, and their structures are complex, often in the form of a ring or a linear structure [14,16]. Plant mitogenomes contain about 30–50 genes, which mainly encode proteins related to energy metabolism, such as respiratory chain complexes, ribosomal RNA (rRNA), and transfer RNA (tRNA) [14,17]. In addition, plant mitogenomes contain a large number of non-coding and repetitive sequences [16]. These repeat sequences lead to dynamic changes in genome structure through recombination events, further increasing their complexity. Plant mitogenomes are often characterized by highly dynamic structures and extremely low rates of nucleotide substitution. For example, studies in *Angelica dahurica* [18] and *Gossypium* [16] have shown that mitogenomes vary significantly in size and structure across species, mainly due to repeated sequence-mediated recombination events. These dynamic rearrangements result in variability in genome size, but the gene content remains highly conserved.

Intracellular gene transfer (IGT) is one of the important drivers of plant genome evolution [19], which mainly includes chloroplast-to-nucleus (NUPTs), mitochondria-to-nucleus (NUMTs), and chloroplast-to-mitochondria (MTPTs) DNA transfer. These processes not only shape the composition of the nuclear genome but also influence the function and evolution of organelles. In recent years, with the development of high-throughput sequencing technologies, researchers have gained a deeper understanding of the mechanisms and dynamics of IGT and its evolutionary significance, especially in polyploid plants and species with complex genomes [19,20]. Significant progress has been made in these areas. The transfer of chloroplast and mitochondrial DNA to the nuclear genome is a continuous and pervasive process. Studies in *Oryza* and wheat have shown that NUPTs and NUMTs are mostly located in intergenic regions and tend to integrate into gene regulatory regions (e.g., promoters and introns) [20,21]. These exogenous DNA fragments are often accompanied by single-nucleotide polymorphisms (SNPs) and insertion deletions (InDels), leading to open-reading-frame disruption and eventual pseudogenization [22]. Epigenetic regulation plays a key role in this process: intranuclear NUPTs/NUMTs, similar to transposons, exhibit high DNA methylation and low-activity histone modifications, which inhibit transcription. In addition, polyploidization events exacerbate subgenomic asymmetries. For example, the subgenome of hexaploid wheat is more prone to accumulate NUPTs/NUMTs due to active transposons and double-strand breaks, which may be related to the synergistic evolution of the maternal cytoplasmic and nuclear genomes [19].

IGT and mitogenome studies are important for the evolution of the *Adansonia* genome and can further reveal the complex picture of plant genome evolution. Currently, the mitogenome of *Adansonia* remains unreported, and this study will provide complete mitogenome data for four *Adansonia* species (*A. digitata*, *A. za, A. perrieri*, and *A. rubrostipa*), laying the foundation for subsequent comparative genomics and evolutionary biology studies. By analyzing the sequence features, gene composition, and structural variation of the mitogenomes of these species, researchers can reveal their evolutionary relationships with other closely related species, reconstruct the phylogenetic framework of the genus, and explore the mechanisms of their genome dynamics during the evolutionary process. In addition, the study of IGT events between the mitochondrial and nuclear genomes will help to understand the co-evolutionary process of the mitochondrial and nuclear genomes and the contribution of gene transfer to the adaptive evolution of the species. These studies will not only deepen the understanding of the biological characteristics of species in the genus *Adansonia* but also provide new perspectives for revealing the evolutionary patterns and functional diversity of plant mitogenomes, which are of great theoretical significance and applied value.

## 2. Materials and Methods

### 2.1. Genome Sequencing Data Collection

Whole-genome sequencing was not performed for the four *Adansonia* species (*A. digitata*, *A. za*, *A. perrieri*, and *A. rubrostipa*) in this study. The MGISEQ short-read and PacBio long-read genome sequencing data were primarily based on a previously published article by Wan et al. 2024 [5] and were downloaded from the NGDC database (https://ngdc.cncb.ac.cn/gsa/browse/CRA015211, accessed on 2 March 2025). Nuclear genome assembly and annotation data [5] were obtained from the Figshare database (https://doi.org/10.6084/m9.figshare.25422502.v2, accessed on 2 March 2025). We also obtained the chloroplast genomes of the four *Adansonia* species and the mitogenomes of ten related species from the NCBI nucleotide database. These sequence accession numbers are as follows: *A. digitata* chloroplast (MT052998), *A. za* chloroplast (MT053006), *A. rubrostipa* chloroplast (MT053004), *A. perrieri* chloroplast (MT053003), *Abelmoschus esculentus* (OL348387–OL348388), *Bombax ceiba* (NC_038052), *Corchorus capsularis* (NC_031359), *Firmiana kwangsiensis* (PP963503), *Gossypium arboreum* (NC_035073), *Hibiscus cannabinus* (NC_035549), *Microcos paniculata* (PP035772), *Theobroma cacao* (NC_066894), *Tilia amurensis* (PQ072837), and *Vatica mangachapoi* (PP861159).

### 2.2. Assembly and Annotation of Mitogenome

To obtain high-quality data, we first processed the raw data using fastp v.0.23.4 [23] to obtain clean reads. Next, we conducted an initial assembly of the mitogenome using GetOrganelle v.1.7.7 [24] with the parameters ‘-R 50 -k 21,45,65,85,105,127 -F emplant_mt’. From the GFA file generated by the assembly, we used Python tools to remove large chloroplast fragments and obtained a mitochondrial draft sequence. Additionally, we mapped the mitogenome draft sequence to PacBio long reads using minimap2 [25]. Subsequently, we extracted the mapped reads using samtools v.1.7 [26] and assembled them using Flye v.2.9 [27]. After the assembly was finished, the final assembly result was generated based on the GFA file with the assistance of Bandage [28].

After completing the assembly of the mitogenome, we comprehensively annotated it using PMGA [29] (http://47.96.249.172:16084/home, accessed on 5 April 2025) and Geneious Prime v.2021.2.2 [30]. Finally, we used PMGmap (http://47.96.249.172:16086/home/, accessed on 5 April 2025) to generate a clear map of the mitogenome.

### 2.3. Repeats Identification and SSRs Analysis

Simple sequence repeats (SSRs) were identified using MISA [31] (https://webblast.ipk-gatersleben.de/misa/, accessed on 5 April 2025) with the following minimum repeat thresholds: 10 for mono-, 5 for di-, 4 for tri-, and 3 for tetra-, penta-, and hexa-nucleotides. Additionally, repfind.pl [32] were employed to detect dispersed repeats, with a minimum size of 8 bp.

### 2.4. Gene Rearrangement and RSCU Analysis

We analyzed the mitogenomes of *A. digitata*, *A. perrieri*, *A. rubrostipa,* and *A. za* for covariance using AliTV [33]. We then analyzed the mitogenomes of four *Adansonia* species and nine closely related species for rearrangement using Mauve. Relative synonymous codon usage (RSCU) was analyzed using PhyloSuite v.1.2.3 [34].

### 2.5. Selective Pressure Analysis

This study used KaKs_Calculator v.2.0 [35] to assess the selective pressure on protein-coding genes (PCGs) in plant mitogenomes, with the primary model being YN, while also employing MLWL, GY, and MA models for cross-validation. Based on the complete mitogenomes of four *Adansonia* species and ten closely related species, PhyloSuite v.1.2.3 [34] was used to extract the nucleotide sequences of 34 core PCGs. Using a Python program, stop codons were removed, and the corresponding protein sequences were converted. We also performed pairwise alignments for the four *Adansonia* species and ten closely related species. Subsequently, ParaAT v.2.0 [36] was used for batch calculations of the *K*a/*K*s values for each pair. Finally, TBtools v.2.0 [37] was used for data visualization. All analyses were based on codon-level alignments to ensure the accuracy of non-synonymous substitution (*K*a) and synonymous substitution (*K*s) calculations.

### 2.6. Analysis of Intracellular Gene Transfer Between Organelles and the Nuclear Genome

This study used BLAST v.2.15.0 software to identify chloroplast-to-mitochondrial transfer sequences (MTPTs), chloroplast-to-nucleus transfer sequences (NUPTs), and mitochondrial-to-nucleus transfer sequences (NUMTs), with an e-value set to 1 × 10^5^. Specifically, MTPTs were obtained by aligning the chloroplast genome with the corresponding mitogenome, NUMTs were obtained by aligning the mitogenome with the nuclear genome, while NUPTs were identified by aligning the chloroplast genome with the nuclear genome. In the IGT detection, false BLAST alignment results caused by low-complexity regions and repetitive elements may misinterpret these non-genuine similarities as evidence of gene transfer. In order to screen for high-confidence insertion sequences, we manually filtered out regions of the genome that were rich in repetitive sequences and low-complexity sequences. In addition, we only selected sequences located on chromosomes, with a sequence length of at least 100 bp and a similarity of at least 80%. Based on sequence similarity, these insertions were classified into two categories: young sequences (similarity ≥ 90%) and ancient sequences (80% ≤ similarity < 90%) [16]. The potential transferred DNA sequences were extracted by their genomic position and analyzed, and further visualized using Circoletto [38] and NGenomeSyn [39].

### 2.7. Phylogenetic Tree Construction

To study the phylogenetic relationships of the *Adansonia* genus, 14 different plant mitochondrial genomes were used, with *Vatica mangachapoi* as the outgroup. We used the PhyloSuite v.1.2.3 [34] to extract common PCGs from the mitogenomes of these species. Each gene was then aligned using MAFFT v.7.4 [40]. After the manual corrections, the aligned sequences for each species were further concatenated using PhyloSuite v.1.2.3 [34]. Based on the matrix of concatenated sequences, the maximum-likelihood (ML) tree was constructed using IQ-TREE v.2.1.2 [41], with the most suitable model and bootstrap value set to TVM + F + I + G4 and 1000, respectively. Tree visualization was achieved in Figtree v.1.4.3 (https://github.com/rambaut/figtree/releases, accessed on 11 April 2025).

## 3. Results and Discussion

### 3.1. Mitogenome Characterization of Adansonia

**Figure 1:**
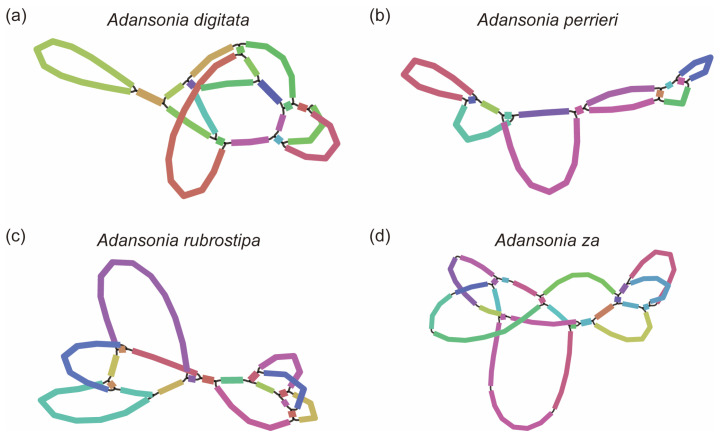
The mitogenomes of *A. digitata*, *A. perrieri*, *A. rubrostipa* and *A. za*. The topological structures of the mitochondrial contigs displayed in Bandage. Different line segments represent different contigs.

**Figure 2:**
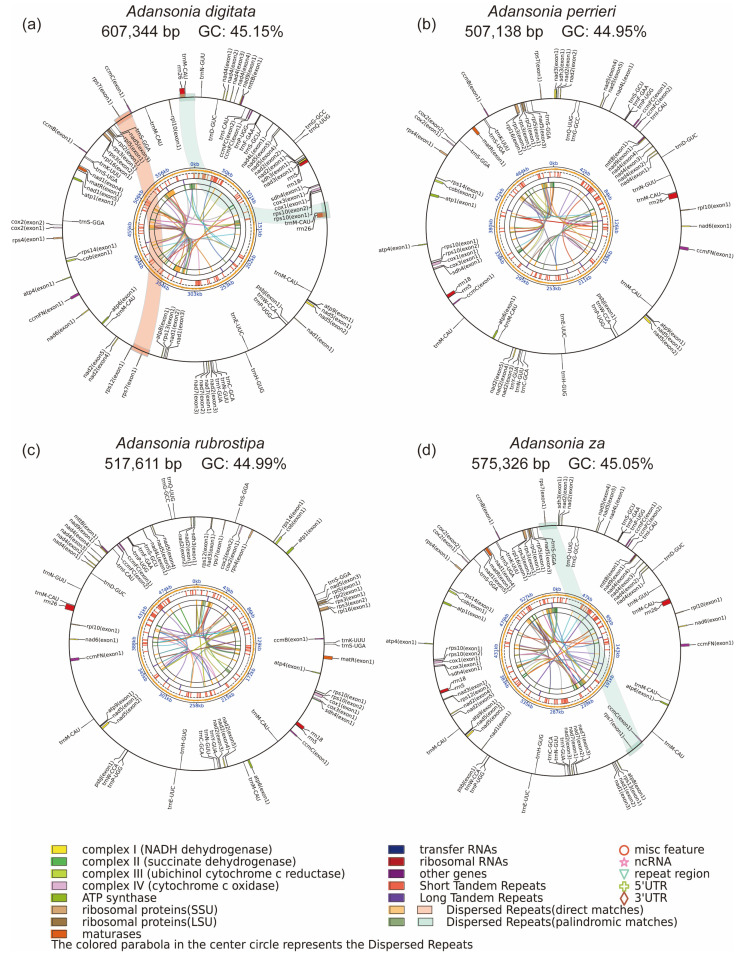
The mitogenomes map of *A. digitata*, *A. perrieri*, *A. rubrostipa* and *A. za*. The inner circle shows the collinear relationship between tandem repeat sequences, while the outer circle shows the collinear region of scattered repeat sequences.

By integrating short-read and long-read data, we successfully assembled the mitogenomes of four *Adansonia* species, which exhibit a complex network structure (Figure 1), with an average read coverage depth ranging from 94.4× to 113.4× (Table 1). These mitogenomes were resolved into single circular continuous sequences ranging in size from 507,138 to 607,344 bp, with GC content ranging from 44.95% to 45.15% (Figure 2, Table 1). However, the structural characteristics of plant mitogenomes are dynamic, and their propensity for recombination makes them prone to subgenomic forms and widespread variation [42]. Unlike the main ring observed in chloroplast and animal mitogenomes, this structure is not the primary form of mitochondrial DNA in plants, with significant variation occurring both within and between species [43,44]. Therefore, further analysis is required to determine the true mitochondrial molecular structure of *Adansonia* species. The variation in size among these genomes may be attributed to factors such as the presence of different amounts of non-coding DNA, gene duplications, insertions, and deletions. The relatively stable GC content within this narrow range may suggest that the mitogenomes of these *Adansonia* species have certain evolutionary constraints in terms of nucleotide composition, which could be related to the stability and function of the encoded genes.

The mitogenomes of the four *Adansonia* species share a similar gene composition, which can be classified into 13 categories (Table S1): NADH dehydrogenase genes (*nad1*, *nad2*, *nad3*, *nad4*, *nad4L*, *nad5*, *nad6*, *nad7*, *nad9*), cytochrome c biogenesis (*ccmB*, *ccmC*, *ccmFC*, *ccmFN*), succinate dehydrogenase genes (*sdh3*, *sdh4*), ubiquinol cytochrome c reductase gene (*cob*), cytochrome c oxidase genes (*cox1*, *cox2*, *cox3*), ATP synthase genes (*atp1*, *atp4*, *atp6*, *atp8*, *atp9*), ribosomal protein small subunit genes (*rps10*, *rps12*, *rps13*, *rps14*, *rps3*, *rps4*, *rps7*), ribosomal protein large subunit genes (*rpl5*, *rpl10*, *rpl16*), membrane transport protein-encoding genes (*mttB*), mature enzyme-encoding genes (*matR*), tRNA genes, rRNA genes (rrn5, rrn18, rrn26), and other genes (Table S1). The gene numbers in the mitogenomes of the four species vary: *A. digitata* has 66 genes (39 PCGs, 24 tRNAs, and 3 rRNAs); *A. perrieri* has 59 genes (33 PCGs, 23 tRNAs, and 3 rRNAs); *A. rubrostipa* has 60 genes (34 PCGs, 23 tRNAs, and 3 rRNAs); and *A. za* has 65 genes (39 PCGs, 23 tRNAs, and 3 rRNAs) (Table 1). Consistent with most species, the mitogenomes of the four *Adansonia* species exhibit low gene density, which may be related to their frequent structural rearrangements. Compared to chloroplast genomes, mitogenomes exhibit higher variability in size, gene number, and arrangement, but their gene content is relatively conserved. Graph-based assembly revealed the complex conformation of the mitogenomes of Malvaceae plants, consistent with previous findings in other Malvaceae species [45,46,47], indicating that large repetitive sequences significantly influence mitogenome structural variation. Additionally, the mitogenomes of these four species all retain the *psbJ* gene derived from the chloroplast genomes, which may be of significant importance for the evolution of *Adansonia* species (Table S1).

### 3.2. Repeats and Collinear Analysis in Four Adansonia Mitogenomes

**Figure 3:**
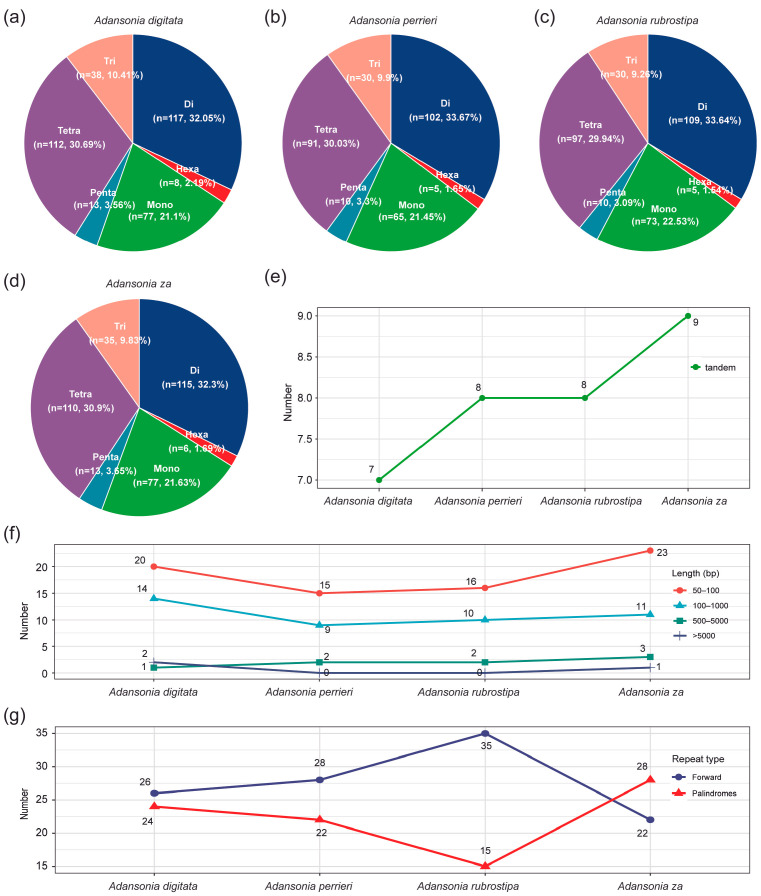
Repeat sequence analysis of the four *Adansonia* mitogenomes. (**a**–**d**) Number and percentage of SSRs in the four *Adansonia* mitogenomes. Mono, di, tri, tetra, penta, and hexa represented mononucleotide, dinucleotide, trinucleotide, tetranucleotide, pentanucleotide, and hexanucleotide, respectively. (**e**) Number of tandem repeats in the four *Adansonia* mitogenomes. (**f**) Statistics on the number of repeats in different length intervals. (**g**) Number of dispersed repeats.

SSRs (Simple sequence repeats) have widespread applications in molecular marking, phylogenetic comparison, genetic diversity analysis, variety identification, and breeding research [48]. This study conducted SSR analysis on the mitogenomes of four *Adansonia* species. The results showed that the number of SSR loci ranged from 303 (*A. perrieri*) to 365 (*A. digitata*), covering six SSR types. Among these, dinucleotide repeats had the highest proportion, ranging from 32.05% to 33.66%; tetranucleotide repeats were the second highest, accounting for 29.94% to 30.90%; mononucleotide repeats also accounted for a significant proportion, ranging from 21.10% to 22.53%; while hexanucleotide repeats had the lowest proportion, only 1.54% to 2.19% (Figure 3a–d). Additionally, the number of tandem repeat sequences in the mitogenomes of the four *Adansonia* species ranged from 7 to 9 (Table 2), with lengths ranging from 2 to 29 bp. Specifically, 7 tandem repeat sequences were identified in *A. digitata*, 8 each in *A. perrieri* and *A. rubrostipa*, and 9 in *A. za* (Figure 3e). Among these, the copy number of tandem repeat sequences with a length of 2 bp was the highest, reaching 18-fold; while the copy number of tandem repeat sequences with a length of 29 bp was also relatively high, ranging from 2.3 to 3.3-fold (Table 2). After comparing the mitogenome repeat sequences of four *Adansonia* species, it was found that repeat sequences ranging in length from 50 to 100 bp were the most abundant (Figure 3f). Among the dispersed repeat types, forward repeats and palindromic repeats were relatively abundant. However, *A. rubrostipa* exhibited a unique situation, with the highest number of forward repeats and the lowest number of palindromic repeats (Figure 3g).

**Figure 4:**
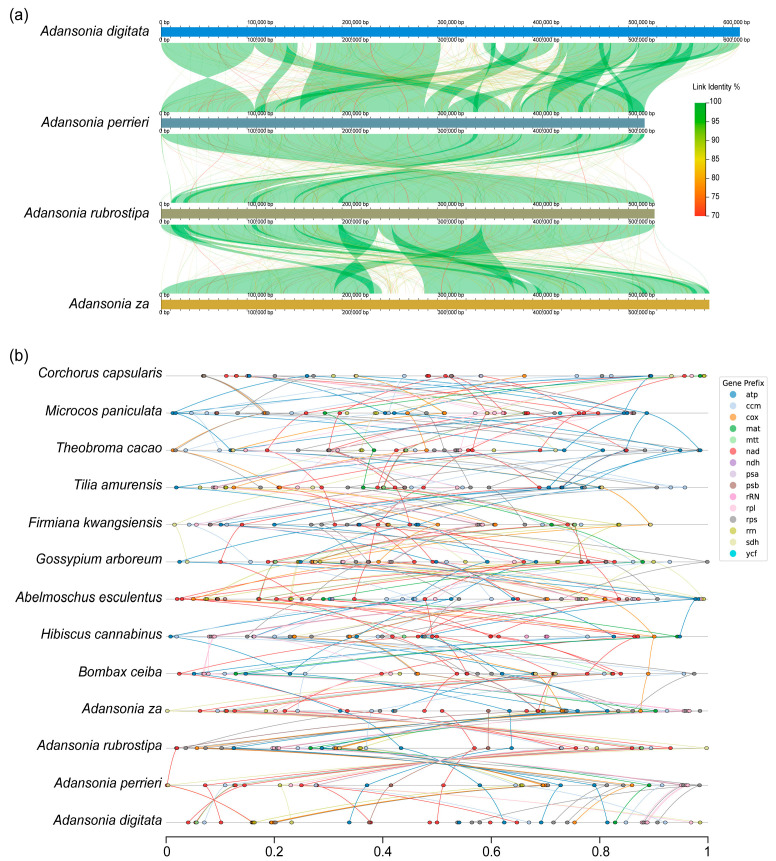
Genomic synteny comparison analysis. (**a**) Collinearity analysis between the four *Adansonia* mitogenomes. (**b**) Gene rearrangement analysis between the four *Adansonia* and other species mitogenomes.

Repeat sequences are one of the common features of genomes. Notably, the amplification of repeat sequences often leads to changes in genome size, a phenomenon that is frequently used to explain the observed diversity in the size and structure of mitogenomes [14,49]. Collinearity analysis revealed that the mitogenomes of the four *Adansonia* species exhibit high similarity, indicating that the mitogenomes of closely related species share a degree of conservation (Figure 4a). However, rearrangements in mitogenomes are relatively common, and significant gene rearrangements are present in the mitogenomes of these four *Adansonia* species (Figure 4b). Compared to chloroplast genomes, the structural stability of plant mitogenomes is significantly lower. This difference primarily stems from the higher content of repetitive sequences in mitogenomes and their complex structural organization [14,50,51].

### 3.3. Relative Synonymous Codon Usage and Select Pressure Analysis for PCGs

**Figure 5:**
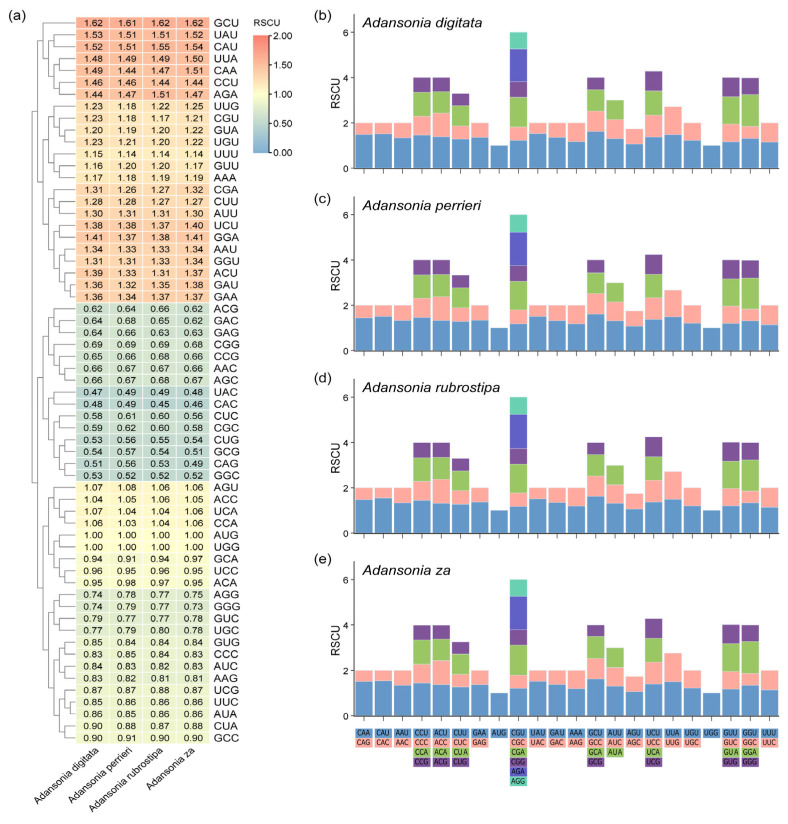
Relative synonymous codon usage (RSCU) analysis. (**a**) RSCU value of four *Adansonia* mtPCGs. (**b**–**e**) Determination of RSCU of four *Adansonia* mtPCGs. The *X*-axis showed the different kinds of codon families for each amino acid. RSCU values represent the frequency of a specific codon in comparison to the uniform synonymous codon usage.

In gene sequences, the use of codons often exhibits bias, which may influence gene expression [52,53]. To investigate the codon preference characteristics of PCGs in the mitogenomes of four *Adansonia* species, we conducted a relative synonymous codon usage (RSCU) analysis (Figure 5). When the RSCU value of a codon exceeds 1, it indicates that the codon is preferred for amino acid synthesis (Table S2). Notably, except for the start codon AUG and the tryptophan codon UGG, which have fixed RSCU values of 1, the codon usage preferences of mtPCGs exhibit distinct patterns. The analysis revealed that a total of 24 codons (GCU, UAU, CAU, UUA, CAA, CCU, AGA, UUG, CGU, GUA, UGU, UUU, GUU, AAA, CGA, CUU, AUU, UCU, GGA, AAU, GGU, ACU, GAU, GAA) have RSCU values greater than 1.0 (Figure 5a). This phenomenon of codon usage preference is widely observed in the mitochondria of most species. Among the four *Adansonia* plants, the most frequently used codons are GCU (corresponding to alanine Ala), UAU (corresponding to tyrosine Tyr), and CAU (corresponding to histidine His), with RSCU values exceeding 1.5.

**Figure 6:**
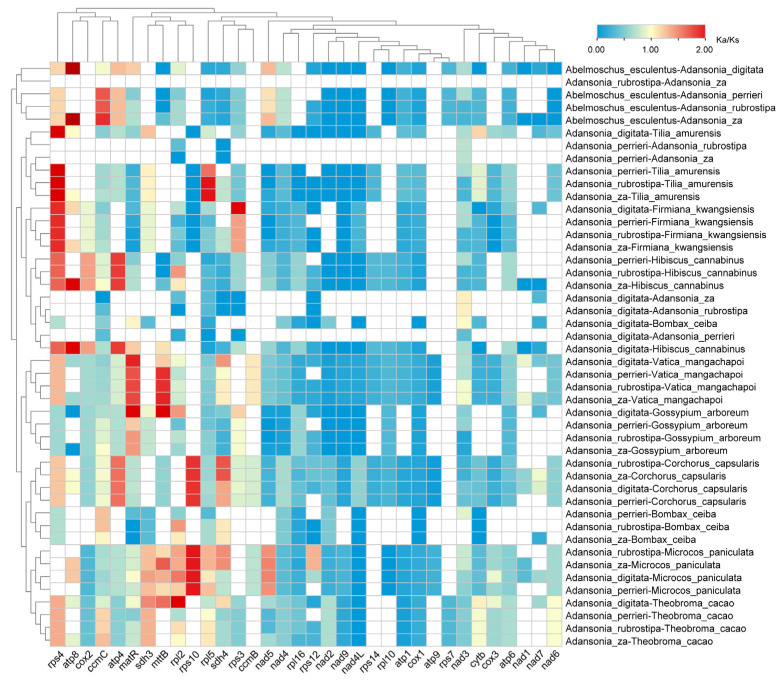
*K*a/*K*s analysis of *Adansonia* and other species. White indicates a value of NA.

To assess the evolutionary rate of mtPCGs, we conducted a selective pressure analysis on 34 PCGs in the mitogenomes of the four *Adansonia* species and other Malvaceae plants, which resulted in 46 comparison combinations (Figure 6). The results showed that the *K*a/*K*s values of most mtPCGs were less than 1, indicating that these genes underwent purifying selection during evolution. Additionally, when comparing *Adansonia* with *Abelmoschus*, *Tilia*, *Firmiana*, *Hibiscus*, *Vatica*, *Corchorus*, and *Theobroma*, the *K*a/*K*s value of the *rps4* gene was found to be greater than 1 (Figure 6), suggesting that this gene may have undergone positive selection during the evolution of *Adansonia* and may play a role in environmental adaptation. However, further analysis is needed to confirm this conclusion. In addition to *rps4*, the *atp8*, *cox2*, *ccmC*, *atp4*, *matR*, *sdh3*, *mttB*, *rpl2*, *rps10*, *rpl5*, *sdh4*, *rps3*, and *nad5* genes also showed signs of potential positive selection in certain comparison combinations (Figure 6). In contrast, no evidence of positive selection was detected in the four *Adansonia* species, suggesting that mtPCGs in the *Adansonia* genus are highly conserved.

### 3.4. Intracellular Gene Transfer Analysis

**Figure 7:**
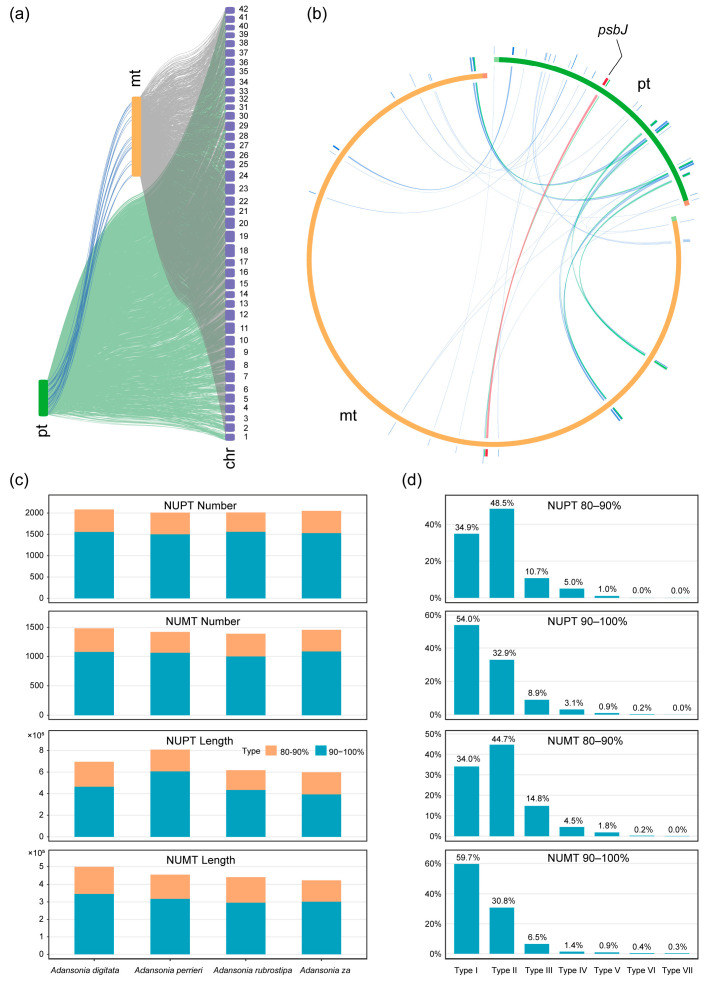
Intercellular gene transfer analysis of four *Adansonia* species. (**a**) Schematic diagram of chloroplast DNA fragments transferred into the mitogenome. The red line represented a transferred DNA fragment having 100% similarity, the orange line represented a DNA fragment with 75–99% similarity, the green line represented a DNA fragment with 50–75% similarity, and the blue line represented a DNA fragment similarity less than 50%. (**b**) Distribution characteristics of NUOTs and MTPTs in the *Adansonia* genome. (**c**) Characteristics of recent and ancient NUOTs in four *Adansonia* species. (**d**) Distribution of seven types of NUOTs in four *Adansonia* species.

**Figure 8:**
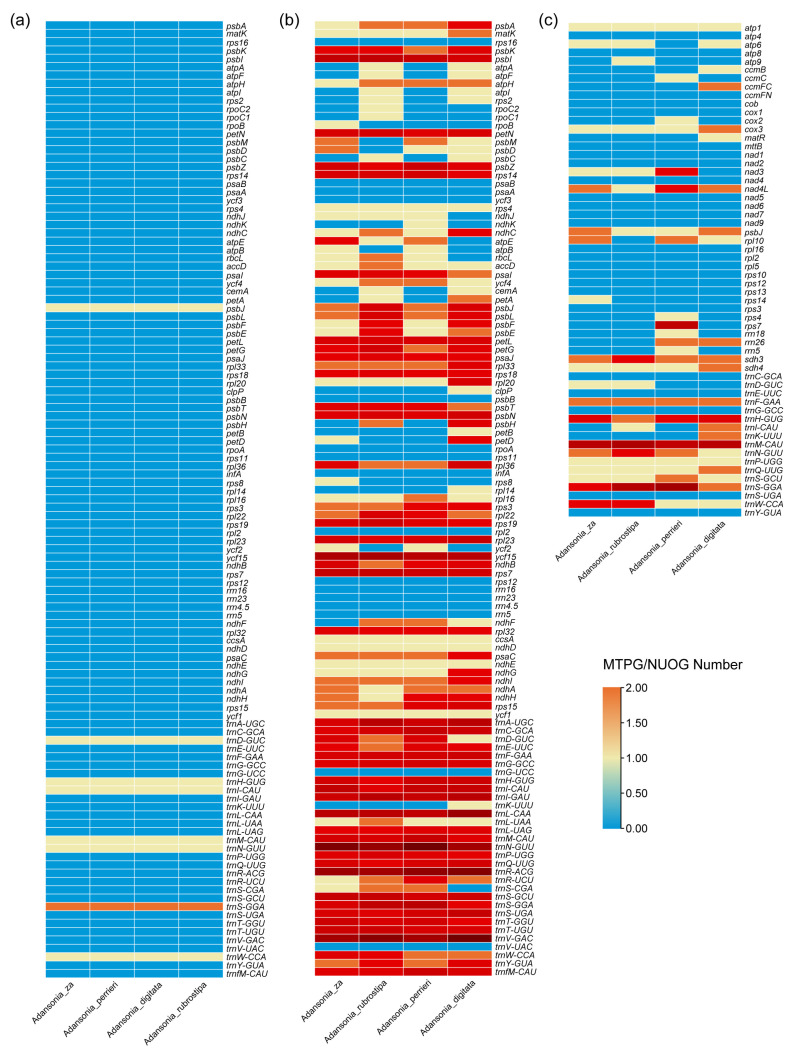
Statistical count of MTPGs/NUOGs in four *Adansonia* species. (**a**) Statistical count of MTPGs. (**b**) Statistical count of NUPGs. (**c**) Statistical count of NUMGs.

In the plant kingdom, intercellular gene transfer (IGT) frequently occurs between the nuclear genome, chloroplast genome, and mitogenome [22]. To investigate the IGT patterns among these three genomes in four *Adansonia* species, we identified the three gene transfer pathways: chloroplast to mitochondria, chloroplast to nucleus, and mitochondria to nucleus. In the mitogenomes of the four *Adansonia* species, transfer sequences originating from the chloroplast genome were identified (Figure 7b). These transfer sequences primarily originate from non-coding regions of the chloroplast genome, while coding regions mainly include tRNA genes, rRNA sequences, and chloroplast-encoded genes. Additionally, most transfer sequences have not undergone significant changes. Sequence similarity analysis revealed 22–29 homologous fragments between the two organelle genomes, ranging in length from 30 bp to 1501 bp (Table S4). Previous studies have indicated that tRNA has a high probability of transfer between higher plant genomes and can randomly insert into the genome [54]. Annotation results indicated that 7 homologous segments are complete tRNA genes (*trnD-GUC*, *trnH-GUG*, *trnI-CAU*, *trnM-CAU*, *trnN-GUU*, *trnS-GGA*, *trnW-CCA*), and rrn16 is an incomplete rRNA gene (Figure 8a; Table S4). Notably, the rRNA genes that migrated from chloroplasts to mitochondria underwent certain changes, which may be related to the functional roles of tRNA and rRNA in biological processes. Compared to rRNA, biological processes place higher demands on the functional conservation of tRNA. Additionally, the *psbJ* gene of chloroplast origin was identified in the mitogenome (Figure 7b; Table S4).

Regarding the sequences transferred from the mitochondrial and chloroplast genomes to the nuclear genome, the results show that the chloroplast and mitogenomes can fully transfer to the nuclear genome and are distributed across the chromosomes of the nuclear genomes of the four *Adansonia* species (Figure 7a; Tables S4 and S5). We classified nuclear organelle DNA transfer sequences (NUOTs) based on sequence similarity, defining those with similarity between 90–100% as recent types and those with similarity between 80–90% as ancient types [16]. This classification helps to roughly estimate the insertion time of these sequences. By analyzing the distribution of transfer sequences, we speculate that organelle DNA was gradually transferred into the nuclear genome during evolution, with frequent insertions occurring recently. Notably, the number of recently inserted sequences typically exceeds that of ancient sequences (Figure 7c). This may be due to the degradation of ancient NUOTs over time, making their sequence characteristics increasingly difficult to identify and thereby reducing detection accuracy [22]. Among the four *Adansonia* species, the number and length of NUOTs vary significantly, showing no obvious conservation. Overall, the number of nuclear-encoded chloroplast DNA transfer sequences (NUPTs) is generally higher than that of nuclear-encoded mitochondrial DNA transfer sequences (NUMTs), and their lengths are relatively longer (Figure 7c). Statistical analysis of different types of NUOTs revealed that both recent and ancient NUOTs exhibit a higher abundance of Type I and Type II sequences, with fewer sequences of other types (Figure 7d). This result suggests that NUOTs tend to be preserved in short fragment forms, possibly because short NUOTs cause less disruption to the original structure and function of the nuclear genome and are more easily integrated into the regulatory network of the nuclear genome. Additionally, we screened NUOTs to identify nuclear organelle genes (NUOGs, i.e., NUPGs and NUMGs) with complete protein-coding regions, all of which were found in recently inserted sequence types. However, these genes account for only a small portion of NUOTs, possibly because organelle sequences transferred to the nuclear genome are susceptible to various factors in their new environment, such as gene mutations and recombination, leading to widespread loss of integrity. NUOGs retained in the nuclear genomes of four *Adansonia* species exhibit certain similarities (Figure 8; Tables S4 and S5). Overall, NUPGs have a higher copy number than NUMGs. Among NUPGs, tRNA copies are the most abundant, except for some coding genes, and NUMGs exhibit similar characteristics.

### 3.5. Phylogenetic Analysis

**Figure 9:**
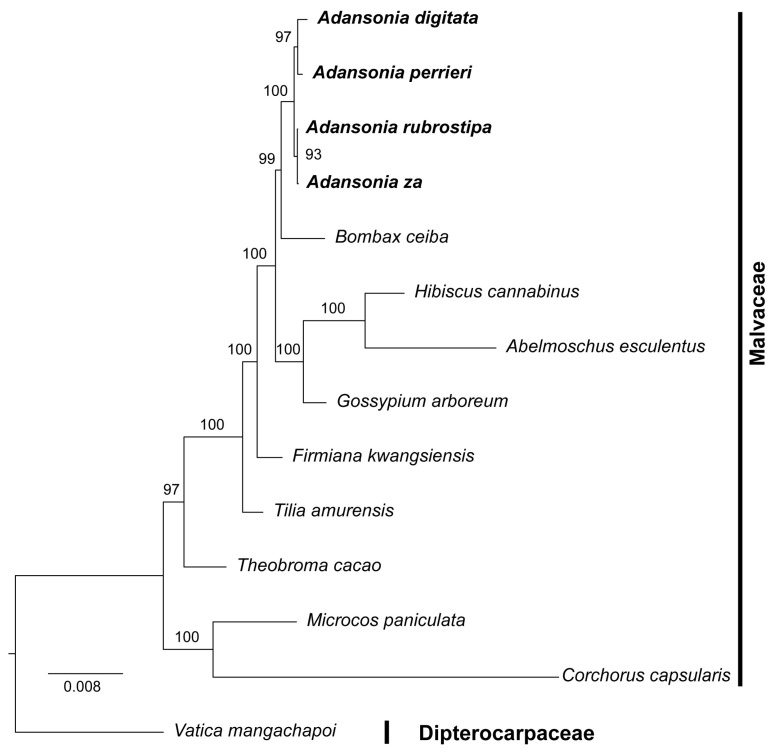
Phylogenetic tree of four *Adansonia* species based on mtPCGs. The numbers on the branches indicated the bootstrap values of the ML tree.

To clarify the phylogenetic position of the *Adansonia* genus within the family Malvaceae, we conducted a phylogenetic analysis of four *Adansonia* species and ten other species. Specifically, we used the maximum likelihood (ML) method to construct a phylogenetic tree, with the data matrix consisting of 34 mtPCGs aligned and concatenated. The phylogenetic tree showed that most branch nodes had high support rates, indicating that the phylogenetic relationships revealed by the mtPCGs are reliable (Figure 9). The phylogenetic analysis showed that the *Adansonia* genus is closely related to the *Bombax* genus, a conclusion consistent with previous studies [5]. However, within the *Adansonia* genus, the mitochondrial phylogenetic tree exhibited different clustering patterns: *A. rubrostipa* and *A. za* clustered closely together, while *A. digitata* and *A. perrieri* formed another cluster (Figure 9). This result of the mitochondrial phylogenetic tree differs significantly from that of the phylogenetic tree constructed based on nuclear genes. Previous phylogenetic trees based on nuclear genes indicated that *A. digitata* is more closely related to *A. rubrostipa*, while *A. za* is more closely related to *A. perrieri* [5]. The inconsistency between the results of the phylogenetic tree based on mitochondrial genes and that based on nuclear genes may suggest complex evolutionary events within the *Adansonia* genus. Mitochondrial and nuclear genes have different genetic mechanisms and evolutionary rates during inheritance. Mitochondrial genes typically follow maternal inheritance and have relatively faster evolutionary rates, making them more susceptible to environmental factors and random genetic drift. In contrast, nuclear genes have more complex inheritance patterns involving parental inheritance and genetic recombination, resulting in relatively stable evolution. These differences in genetic characteristics may lead to divergent results when revealing species relationships. Additionally, historical events such as horizontal gene transfer and hybridization may also influence the evolution of the *Adansonia* genus [55]. Horizontal gene transfer enables species to acquire genes from other species, thereby altering their genetic composition and evolutionary trajectory. Hybridization and introgression may result in gene exchange and fusion between different species, further disrupting the phylogenetic relationships originally constructed based on a single gene type. Although bioinformatics methods for detecting horizontal gene transfer [56,57], hybridization, and introgression [58] currently exist, a comprehensive understanding of these evolutionary processes will require the combination of high-quality nuclear and organellar genome data in the future.

## 4. Conclusions

Thanks to the rapid development of sequencing technology, deciphering the mitogenome sequences of higher plants has gradually become a popular area of research. Compared to chloroplast genomes, mitogenomes are more challenging to assemble, presenting numerous complex challenges to the research process. Additionally, the structure of mitogenomes is highly dynamic, which has contributed to the relatively limited research on higher plant mitochondria to date. The *Adansonia* genus belongs to the family Malvaceae, with a small number of species, and research on the organelle genomes of this genus is particularly scarce. In this study, we conducted an in-depth analysis of the mitogenome sequences of four *Adansonia* species. The results indicate that these mitogenomes can be resolved into a single circular sequence. Although there are significant differences in genome length among the species, the gene content exhibits a high degree of conservation. Further analysis indicated that the mitogenomes of the four *Adansonia* species contain a large number of repeats. Among these, the most frequently occurring SSRs were dinucleotide, tetranucleotide, and mononucleotide repeats. Collinearity and rearrangement analyses showed that the mitogenomes of the four *Adansonia* species exhibited high collinearity, but also significant gene rearrangements. These rearrangements altered the sequences in the intergenic regions, though their impact on gene distribution patterns was relatively minor. Results from selective pressure analysis indicate that the mitogenomes of the four *Adansonia* species are predominantly subject to purifying selection when growing in natural environments. Notably, compared to plants from the genera *Abelmoschus*, *Tilia*, *Firmiana*, *Hibiscus*, *Vatica*, *Corchorus*, and *Theobroma*, the *rps4* gene in *Adansonia* is generally subject to significant positive selection. Phylogenetic analysis clearly revealed that the *Adansonia* genus is more closely related to the *Bombax* genus, but the relationships within the *Adansonia* genus differ from those in the nuclear gene phylogenetic tree. The findings of this study not only enrich the genomic information of the *Adansonia* genus but also make an important contribution to the phylogenetic and evolutionary studies of Malvaceae plants. In the future, we can expand the scope of research to include more *Adansonia* species, thereby comprehensively and deeply understanding the diversity and evolutionary characteristics within the mitogenomes of this genus. Additionally, by combining transcriptomics, proteomics, and other omics technologies, we can investigate the mechanisms by which mitochondrial genes are expressed and the functions of mitochondrial proteins. This will reveal the key role of mitochondria in the growth, development, and environmental adaptation of *Adansonia* plants.

## Figures and Tables

**Table 1:** Statistics of length, GC content, and gene number in four *Adansonia* mitogenomes.

	*Adansonia digitata*	*Adansonia perrieri*	*Adansonia rubrostipa*	*Adansonia za*
Accession	PV591953	PV591954	PV591955	PV591956
Length	607,344	507,138	517,611	575,326
All gene	67	59	60	65
CDS Number	39	33	34	39
rRNA Number	4	3	3	3
tRNA Number	24	23	23	23
GC Content	45.15%	44.95%	44.99%	45.05%
Mean depth	99.2×	113.4×	94.4×	109.8×

**Table 2:** Statistics on tandem repeat sequences of four *Adansonia* species.

Species	Start	End	Period Size	Copy Number	Species	Start	End	Period Size	Copy Number
*Adansonia perrieri*	65,853	65,883	15	2.1	*Adansonia digitata*	32,075	32,104	15	2.1
	117,449	117,516	29	2.3		220,838	220,872	2	18
	169,765	169,791	10	2.7		246,795	246,840	23	2
	203,567	203,601	2	18		341,385	341,420	17	2.1
	229,520	229,565	23	2		429,576	429,606	14	2.2
	301,130	301,179	25	2		549,109	549,144	17	2.1
	407,771	407,801	14	2.2		584,992	585,041	25	2
	497,972	498,007	17	2.1	*Adansonia za*	65,242	65,272	15	2.1
*Adansonia rubrostipa*	20,011	20,046	17	2.1		116,867	116,934	29	2.3
	57,450	57,480	14	2.2		169,164	169,190	10	2.7
	216,871	216,920	25	2		192,505	192,554	25	2
	288,485	288,530	23	2		228,408	228,443	17	2.1
	314,449	314,483	2	18		322,990	323,035	23	2
	348,266	348,292	10	2.7		348,954	348,988	2	18
	400,522	400,618	29	3.3		475,327	475,357	14	2.2
	452,213	452,242	15	2.1		565,540	565,575	17	2.1

## Data Availability

The genomic data that support the findings of this study were available in GenBank of NCBI (https://www.mdpi.com/ethics, accessed on 5 April 2025) under the accession no. PV591953- PV591956. Genomes and raw genome sequencing data are from previously published articles [5].

## Supplementary Materials

The following supporting information can be downloaded at: https://www.mdpi.com/article/10.3390/genes16070846/s1. Table S1: Statistics on mitochondrial gene function. Table S2: RSCU value of 34 PCGs in four *Adansonia* mitogenomes. Table S3. *K*a/*K*s values for 34 genes in a combination of four *Adansonia* species and other Malvaceae species. Table S4. MTPT annotation results for four *Adansonia* species. Table S5. NUPT annotation results for four *Adansonia* species. Table S6. NUMT annotation results for four *Adansonia* species.
